# Non-mitotic proliferation of malignant cancer cells revealed through live-cell imaging of primary and cell-line cultures

**DOI:** 10.1186/s13008-024-00109-x

**Published:** 2024-02-10

**Authors:** Iram Shazia Tyagi, Ho Yin Calvin Tsui, Si Chen, Xinyi Li, Wai-Kin Mat, Muhammad A. Khan, Lucas Brendan Choy, Ka-Yin Aden Chan, Tat-Ming Danny Chan, Chi-Ping Stephanie Ng, Ho-Keung Ng, Wai Sang Poon, Hong Xue

**Affiliations:** 1 Division of Life Science, Hong Kong University of Science and Technology, Clear Water Bay, Hong Kong SAR China; 2 Department of Anatomical and Cellular Pathology, The Chinese University of Hong Kong, Hong Kong, Hong Kong SAR China; 3 Division of Neurosurgery & CUHK Otto Wong Brain Tumour Centre, Department of Surgery, The Chinese University of Hong Kong (CUHK), Hong Kong, Hong Kong SAR China; 4 Department of Neurosurgery, Neuro-Medical Centre, University of Hong Kong-Shenzhen Hospital, Shenzhen, 518053 Guangdong China; 5 Center for Cancer Genomics, School of Basic Medicine and Clinical Pharmacy, China Pharmaceutical University, Nanjing, China; 6 Guangzhou HKUST Fok Ying Tung Research Institute, Science and Technology Building, Nansha Information Technology Park, Nansha, 511458 Guangzhou China

**Keywords:** Amitosis, Anti-mitotic drugs, Cannibalism, Daughter number variation, Endomitosis, Nucleic DNA leakage, Shedding, Tunneling

## Abstract

**Introduction:**

Anti-mitosis has been a key strategy of anti-cancer therapies, targeting at a fundamental property of cancer cells, their non-controllable proliferation due to overactive mitotic divisions. For improved anti-cancer therapies, it is important to find out whether cancer cells can proliferate independent of mitosis and become resistant to anti-mitotic agents.

**Results:**

In this study, live-cell imaging was applied to both primary-cultures of tumor cells, and immortalized cancer cell lines, to detect aberrant proliferations. Cells isolated from various malignant tumors, such as Grade-III hemangiopericytoma, atypical meningioma, and metastatic brain tumor exhibit distinct cellular behaviors, including amoeboid sequestration, tailing, tunneling, nucleic DNA leakage, as well as prokaryote-like division such as binary fission and budding-shedding, which are collectively referred to and reported as ‘non-mitotic proliferation’ in this study. In contrast, benign tumors including Grade-I hemangiopericytoma and meningioma were not obvious in such behaviors. Moreover, when cultured in medium free of any anti-cancer drugs, cells from a recurrent Grade-III hemangiopericytoma that had been subjected to pre-operation adjuvant chemotherapy gradually shifted from non-mitotic proliferation to abnormal mitosis in the form of daughter number variation (DNV) and endomitosis, and eventually regular mitosis. Similarly, when treated with the anti-cancer drugs Epirubicin or Cisplatin, the cancer cell lines HeLa and A549 showed a shift from regular mitosis to abnormal mitosis, and further to non-mitosis as the dominant mode of proliferation with increasing drug concentrations. Upon removal of the drugs, the cells reversed back to regular mitosis with only minor occurrences of abnormal mitosis, accompanied by increased expression of the stem cell markers ALDH1, Sox, Oct4 and Nanog.

**Conclusions:**

The present study revealed that various types of malignant, but not benign, cancer cells exhibited cellular behaviors indicative of non-mitotic proliferation such as binary fission, which was typical of prokaryotic cell division, suggesting cell level atavism. Moreover, reversible transitions through the three modes of proliferation, i.e., mitosis, abnormal mitosis and non-mitosis, were observed when anticancer drug concentrations were grossly increased inducing non-mitosis or decreased favoring mitosis. Potential clinical significance of non-mitotic proliferation in cancer drug resistance and recurrence, and its relationship with cancer stem cells are worthy of further studies.

**Supplementary Information:**

The online version contains supplementary material available at 10.1186/s13008-024-00109-x.

## Introduction

There are several types of cell division displayed by different types of organisms. While most prokaryotes employ binary fission to divide, eukaryotes such as plants and animals typically use mitosis among their somatic cells, or meiosis among their germ cells, to divide. An exception exists in the eukaryote Euglena, which reproduces asexually through binary fission, a form of cell division normally observed in prokaryotes [1].

A prominent feature of cancers pertains to their exceptional ability to unlimited cell division and growth. Accordingly, anti-mitosis has been regarded as an important approach for anti-cancer treatment, achieved through the usage of anti-mitotic drugs that inhibit mitosis cell division. Since cancer cells are able to grow and metastasize through continuous mitotic divisions, they are more sensitive to mitosis inhibition than normal cells. Although anti-mitotic agents have been successful clinically, patient response to them remains highly unpredictable and susceptible to drug resistance. Drug toxicity is also a problem. New generations of anti-mitotic drugs are needed to address these limitations as well as the possible involvement of cell division mechanisms beside mitosis.

The hallmark of cancer cells is their replicative immortality, and extensive research has been directed toward understanding the factors underlying their loss of replicative control. While numerous novel anti-cancer drugs have been found to be effective, studies have revealed tumor heterogeneity to be one of the frequent markers of cancer malignancy [2–5].

In this regard, autophagy a homeostatic mechanism that promotes cell survival through self-digestion of organelles and unfolded proteins, has attracted much attention [6]. While upregulation of autophagy contributed to the spread of tumor in mice, its downregulation could accompany a reduction in cancer malignancy [7]. Endoreplication has been reported as well in various types of cancers. Cells entering the G2/M phase without going through the G1 phase of mitosis can result in the formation of uninuclear tetraploid colon cancer cells with greater viability than the diploid cancer cells [8–10]. Cell cannibalism is another strategy adopted by cancer cells under stress, where it would engulf another live cell into its cytoplasm for degradation [11]. Upregulation of cannibalism was displayed by doxorubicin-treated tumor cells that increased their own survival rates or rate of metastasis after engulfing other cells [12–15]. On the other hand, cancer cell cannibalism could reduce the number of cancer cells in a population, thereby restricting their spread [16].

Tunneling nanotube (TNT) was a novel cellular mechanism proposed to account for cell survival through cell–cell communication via transfers of proteins, organelles, signals and pathogens [17]. Recent findings indicated that cancer cells could acquire new capabilities such as enhanced metabolic plasticity, migratory phenotypes, angiogenic ability and tumor aggressiveness via TNT-like connections [18], exemplified by the stimulation of TNT in pancreatic cancer cells by doxorubicin in a dose-dependent manner, enhancing intercellular drug efflux to achieve drug resistance [19].

Recently, we have reported a daughter number variation (DNV) phenotype, where cancer cells divide into multi-daughters that could fuse together to form multinucleate cells (MNCs) with increased malignancy [20, 21]. DNV was stimulated by acidic pH of 6.4, or treatment with either the anti-cancer drug 5-fluorouracil (5-FU) or the phytochemical wogonin. DNV is a two-stage process, consisting of multi-daughter division in Stage 1, which is followed by fusion between sister or non-sister cells in Stage 2. DNV is therefore a type of abnormal mitosis marked by variable numbers of daughter cells, with different nucleation patterns, including the formation of MNCs, which could greatly increase the genetic diversity and potential malignancy of the cells. The entire DNV process can be monitored using live-cell imaging.

In view of this, the purpose of this study was to enquire into the question of how any deviations observed in malignant and benign tumor cells could impact on the proportion of primary cultured cells of various types of brain tumors, primary and recurrent solitary fibrous tumor /hemangiopericytoma (HPC), typical and atypical meningioma, metastatic brain tumor, as well as two commonly studied cancer cell lines, viz., HeLa cells of cervical origin and A549 cells of lung origin when they were challenged with different concentrations of anti-cancer drugs, and monitored for changes in cell morphology, proliferation-related abnormality, and expressions of stem-cell biomarkers.

## Results

Time-lapse and fluorescent microscopic imaging analyses were performed on primary cell cultures of different types of tumors, including a second-recurrence malignant HPC (Case 1), a primary HPC pathologically diagnosed as Grade1 solitary fibrous tumor (Case 2), three malignant primary meningiomas (Cases 3, 6 and 9), three pathologically benign primary meningiomas (Cases 4, 5 and 8), two recurrent meningioma (Cases 7 and 11), and a metastatic brain tumor originating from a breast cancer (Case 70), all clinically diagnosed with histopathology supports. Given that Case 1 stood out as an aggressively malignant form of cancer due to its twice recurrence while Case 2 was characterized as a benign tumor displaying slow growth characteristics, a comprehensive investigation was conducted on these two cases to elucidate more precisely the contrasts between their malignant and benign properties, along with the detailed studies on the broader range of various types of brain tumors to valid the observation and generalized the conclusions. In parallel, the HeLa and A549 cells were studied in the presence or absence of varied dosages of anti-cancer drugs for changes in cellular behaviors and stem cell marker expressions, to shed lights on underlining mechanisms and significance of non-mitotic proliferation observed in this study. The results obtained with respect to histopathology, proliferation-related cellular behaviors and stem-cell marker expressions were recorded as follows.

### Histopathological findings

**Fig. 1:**
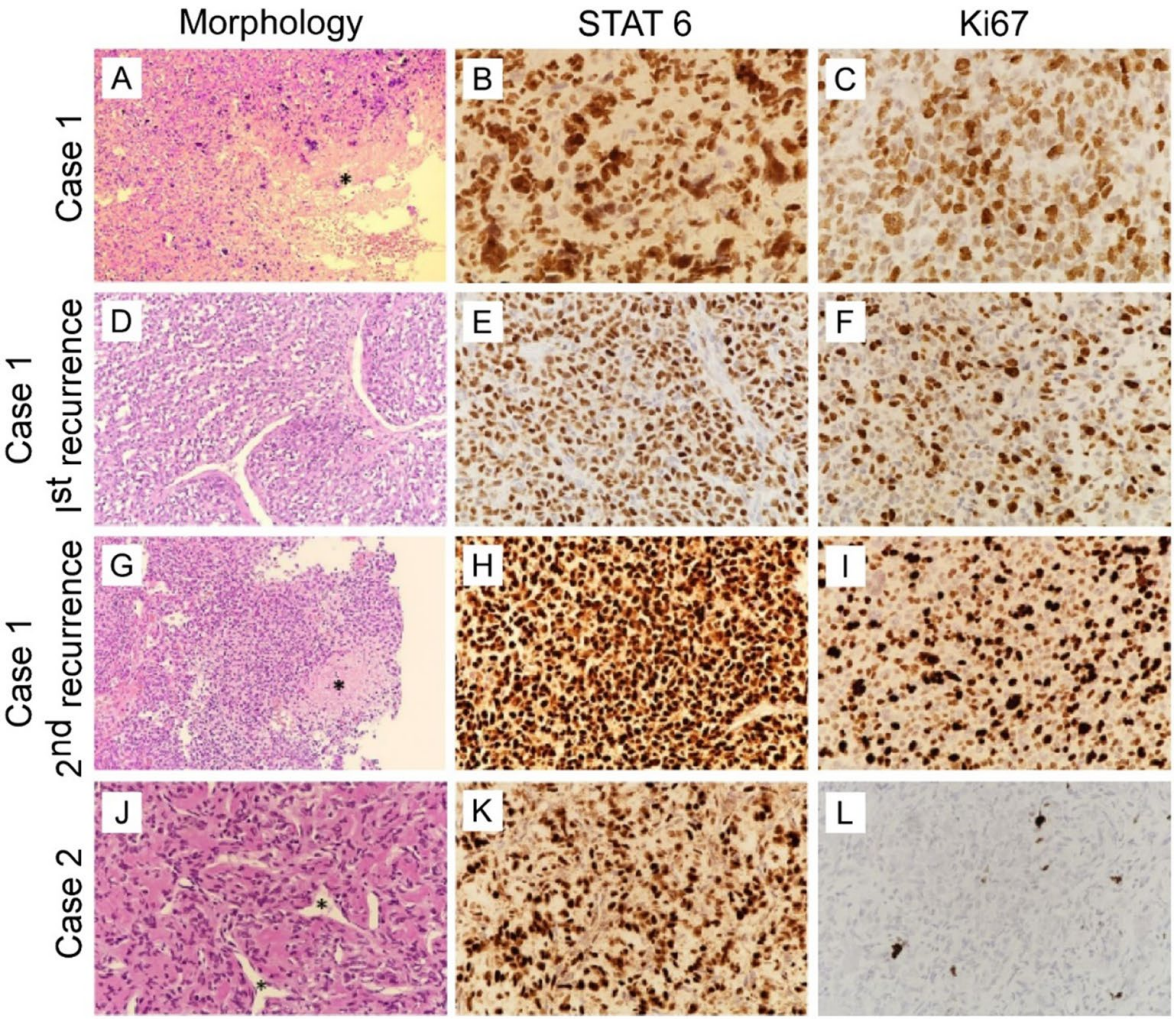
Histopathology of two cases of solitary fibrous tumor /hemangioperictyoma (HPC). Representative snapshots of H&E (**A**, **D**, **G**, 200X and J, 400X), Immuno-staining of marker STAT6 (**B**, **E**, **H**, **K**, 400X) and Ki76 (**C**, **F**, **I**, **L**, 400X). (**A** and **G**) Case 1 primary and second recurrent tumors showing foci of necrosis (asterisk). (**B**, **E**,** H**) STAT6 expression grew higher in the recurrent tumors of Case 1 compared to their previous form. (**J**) Case 2 high power field showed haphazardly arranged spindle cells with moderate cytological atypia, set in collagenous stroma intermixed with staghorn vessels (asterisk), and lack of mitotic figures. (**L**) Case 2 had much lower expression of Ki67 compared to (**C**,** F**,** I**) Case 1. (See more information in Additional file 1: Fig. S1)

When the second-recurrence Case 1 malignant tumor and the benign Case 2 tumor of HPC were subjected to immune-histopathological examination, both of them showed a microscopic tumor with frequent mitosis and some necrosis. To confirm the diagnosis, immunophenotype-staining for STAT6, CD34 and EMA were performed. Case 1 displayed an HPC (WHO Grade III) picture with hyper-cellular and pattern-less sheets of tumor cells, with some foci of necrosis (marked by asterisks in Fig. 1A and Additional file 1: Fig. S1A), and mitotic figures with clear nuclear pleomorphism (Additional file 1: Fig. S1B and S1C). This tumor sample showed positive immunoreactivity for STAT6 and CD34, while EMA was poorly expressed (Fig. 1B, Additional file 1: Fig. S1D and S1E). The proliferation index was very high at 75% upon Ki67 staining (Fig. 1C). Case 1 had experienced cancer relapse twice, each time after two years of surgery. In both recurrences, the tumors exhibited similar histological features of pattern-less architecture with branching blood vessels and diffuse hypercellularity (Fig. 1D and Additional file 1: Fig. S1F, S1G). Mitosis and necrotic foci (marked by asterisks) were also observed (Fig. 1G and Additional file 1: Fig. S1H). Compared to the primary tumor, the recurrent tumors showed a higher Ki67 proliferation rate (Fig. 1F, I) as well as stronger expression of STAT6 (Fig. 1H, K).

The Case 2 cells showed the morphology of an HPC (WHO Grade I), with spindle cells arranged haphazardly with moderate cytological atypia, set in collagenous stroma intermixed with staghorn vessels (asterisks) and few mitotic figures (Fig. 1J and Additional file 1: Fig. S1I). Although the tumor samples showed positive STAT6 expression, EMA and CD34 were found to be negative (Fig. 1K, Additional file 1: Fig. S1J and S1K), and the proliferation index based on Ki67 was low (Fig. 1L).

The results on HPC showed that the Case 1 cells of the second-recurrent malignant tumor expressed Ki67 and STAT6 for more intensely than the primary benign tumor of Case 2. This was in accord with the positive correlation between tumorigenesis and Ki67, a marker of mitotic division activity, with high levels of Ki67 and STAT6 expression that often accompanied high grade relapsed tumors [22–25].

### Higher levels of multi-nucleation in malignant than in benign tumor cells

**Fig. 2:**
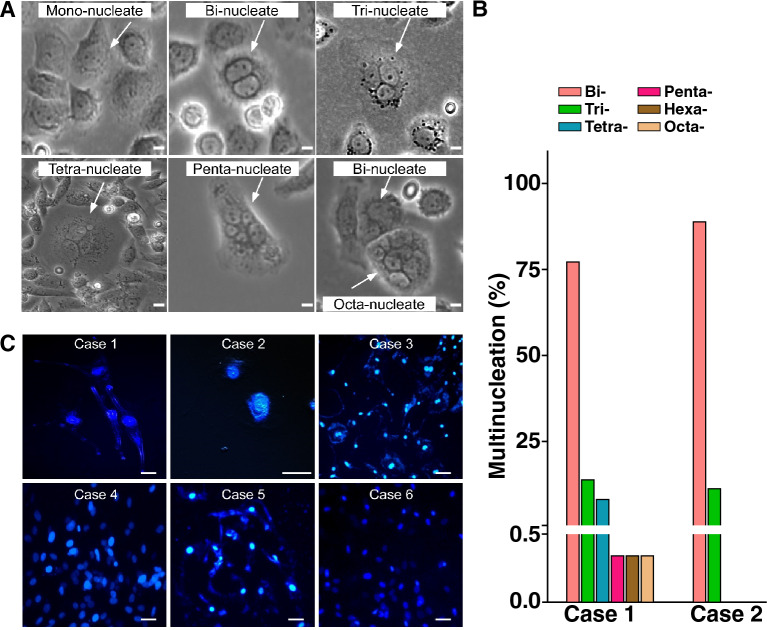
Primary cultured malignant tumor cells showed higher level of multinucleation and nucleic DNA leakage. **A** Selected views of nucleation status of a recurrent HPC tumor from Case 1. Different levels of nucleation were observed through bright-field microscopy. **B** Relative abundance of multi-nucleated cells with various numbers of nuclei, ranging from two (bi-) to eight (octa-) nuclei. Comparison between the malignant Case 1 and benign Case 2, both diagnosed with HPC, showed a higher level of multinucleation in Case 1. **C** Nucleic DNA leakage in primary cultured HPC (Case 1 and 2) and meningioma (Case 3–6). Cells were stained with Hoechst 3324 dye after 15 days of primary culture. The Case 1 and 4 (left two panels) showed obvious loosen nucleus with leakage of nucleic acid materials into cytoplasm. These signs of nucleic DNA leakage were moderate with Case 3 and 6 (right two panels), while nucleus for the benign Case 2 HPC and Case 5 meningioma (middle two panels) were intact and compact with no sign of nucleic DNA leakage. All the scale bars are at 20 µm

When the Case 1 and Case 2 tumor cells were each cultivated as described under Materials and Methods, the Case 1 cells were observable under light microscope within 3 h of culturing, while the cells of Case 2, which originated from a benign tumor showed slower growth, and formed a monolayer only by the eighth day. Under the light microscopy, the Case 1 cells were round and small with a diameter of about 27 µm (Additional file 1: Table S2) with mono-, bi-, tri-, tetra-, penta-, hexa- and octa-nucleate cells (Fig. 2A). The majority of the multinucleate cells were bi-nucleate, accounting for 77% of total MNCs. The tri-nucleate and tetra-nucleate cells amounted to 14% and 8% respectively, while the sum of penta-, hexa- and octa-nucleate cells was 0.34% (Fig. 2B). The Case 2 cells were elongated with a length of approximately 300 µm; and most of them were bi-nucleate with only a small percentage of tri-nucleates.

### Non-mitotic proliferation prominent in primary cultures of malignant tumors

**Fig. 3:**
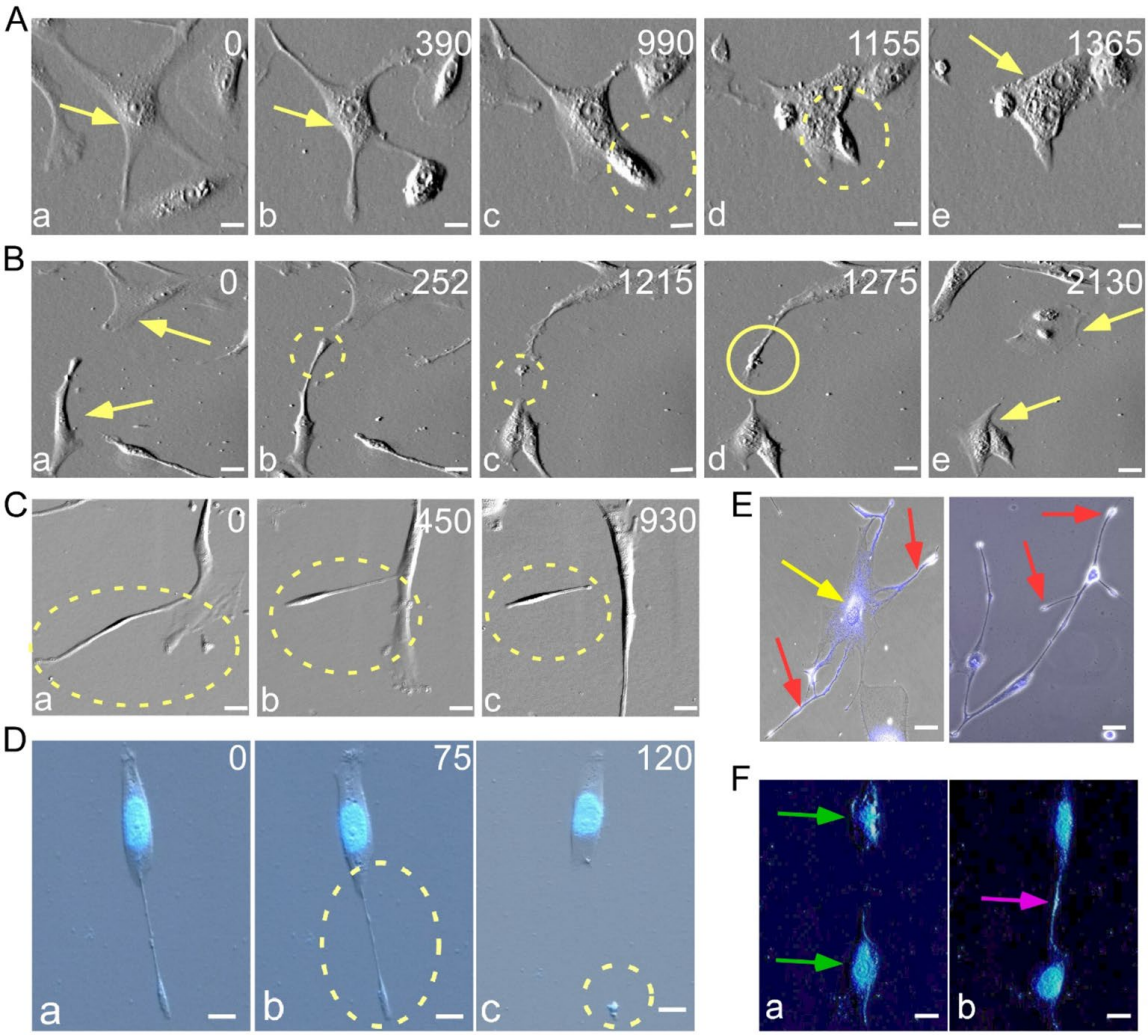
Non-mitotic events observed in primary cultures of the second recurrent HPC tumor from Case 1. **A** Cannibalism through Amoeboid Sequestration: (a) A mononucleate sequestering cell (yellow arrow) adheres to another mononucleate cell. (b) The process of engulfment initiates as the sequestering cell rounds up the target cell. (c-d) Eventually, the target cell gets completely engulfed (dotted circle). (e) This leads to the formation of a single binucleate cell (Additional file 2: Video S1A). **B** Cytoplasmic shedding and acquisition: (a) Two cells (yellow arrows); where (b-c) one of the cells expels a portion of its cytoplasm (dotted circle). (d) The other cell extends itself to incorporate the shed portion (solid circle). (e) Both cells continue to survive after these events (Additional file 3: Video S2B). **C** Protrusion-mediated cell detachment: (a) A cell generates a protrusion from its body. (b) The protrusion narrows from one side. (c) Eventually, the cell detaches from the parent body, achieving independent status (Additional file 3: Video S2A). **D**–**F** Nucleus Staining with Hoechst 33,342. **D** (a) Depicts a cell elongating at one end. (b) This elongation causes the membrane to thin from the center, ultimately resulting in detachment. (c) Through staining, it becomes evident that the shed portion of the parent cell harbors nuclei content (dotted circle) (Additional file 3: Video S2I). **E** Dispersion of nuclear acid materials throughout the cell. Hoechst 33342 staining indicates the presence of nuclear contents not only within the cell nucleus (yellow arrow) but also in other cellular regions (red arrows). **F** Inter-cellular tunneling for nucleic acid transfer: (a) Tunneling is observed between two neighboring cells (green arrows) suggests that (b) the cells are engaged in transferring nucleic acid materials between each other (Additional file 4: Video S3G). All the scale bars are at 50 µm. Time points in minutes are labelled at the upright corner of each panel in (**A**–**D**). The complete videos of these snapshots of live-cell imaging are provided in the Supplementary Materials

After one week of culture, Case 1 cells were monitored by live-cell imaging for seven days. Within this period, cell length extended from 27 to 330 µm (Additional file 1: Table S2). They displayed cannibalism with amoeboid sequestration of other cells or cell debris (Fig. 3A, Additional file 2: Video S1A and B). Some cells underwent shedding of fragments of their cell body which could either remain suspended in the growth medium (Fig. 3C, Additional file 3: Video S2A) or became engulfed by other cells (Fig. 3B, Additional file 3: Video S2B). Cell staining with Hoechst 33342 revealed leaking of nucleic DNA and formation of tunneling nanotubes (Fig. 3D, Additional file 3: Video S2I). Some cells displayed nucleic acid material in different parts of the cytoplasm away from the core nucleus (Fig. 3E, Additional file 4: Video S3H); and many of them were elongated with ‘tails’ and ‘branches’ protruding from the cell body to form nano-tunnels that connected with other cells (Fig. 3F, Additional file 4: Video S3G). Sometimes transfer of nuclei content does not rely on tunneling, in this case, two cells come close to each other and transfer their entire nucleus to each other (Additional file 2: Video S1D). In a few instances, a special type of cell division was observed, where the cell did not round up as usual upon entering into mitosis, but nonetheless succeeded in yielding two daughter cells in some form of binary fission, or amitosis (Additional file 5: Video S4A). Other cells contained particles of cellular material that moved around the cytoplasm and popping in and out of the parent cell (Additional file 4: Video S3A).

**Fig. 4:**
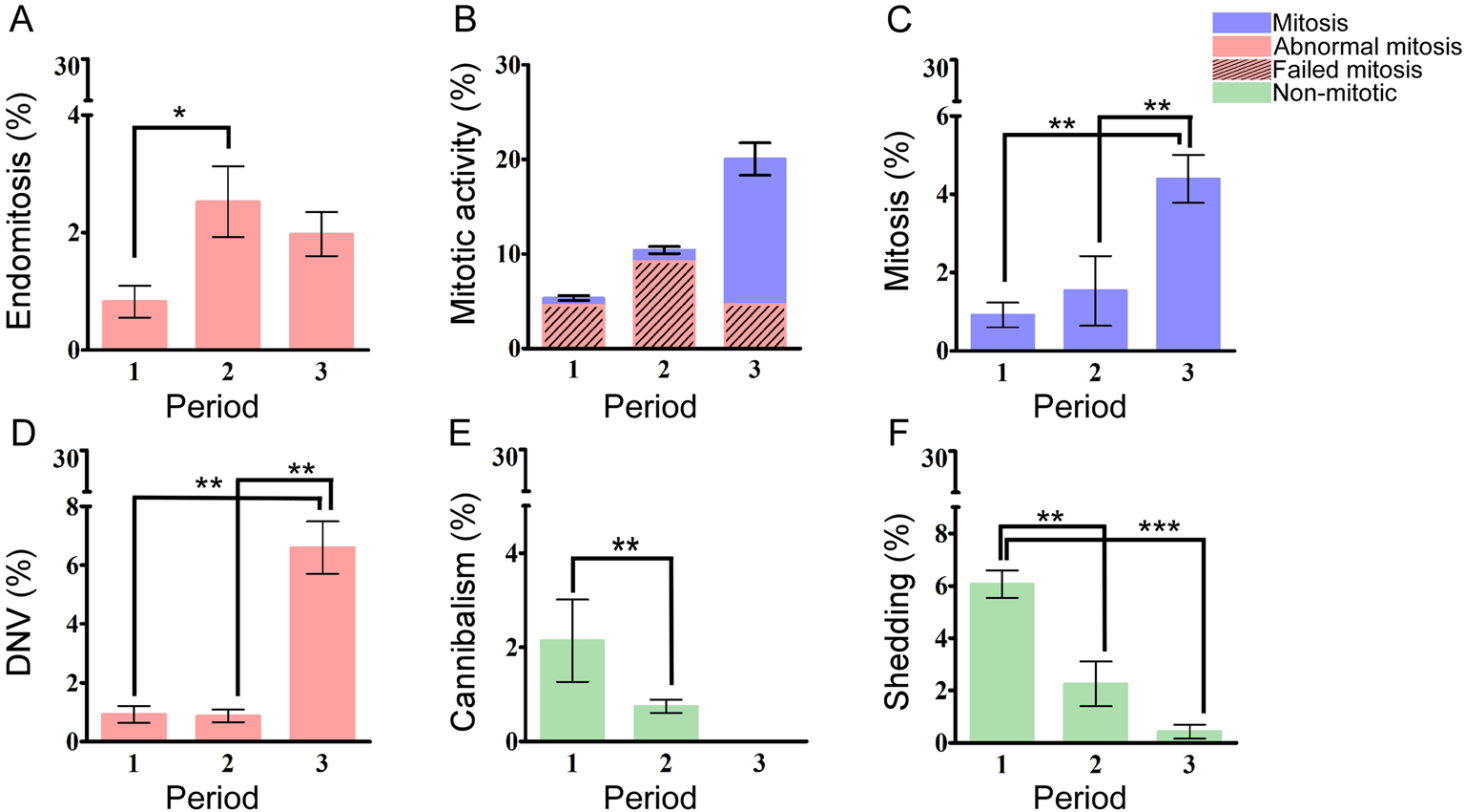
Recovery of mitosis from non-mitotic and abnormal mitotic modes of proliferation in primary cell cultures of the second recurrent HPC tumor from Case 1. The primary culture of the second recurrent tumor of Case 1 was maintained over Period 1 (Day 7–9), Period 2 (Day 10–13) and Period 3 (Day 18). A 24-h live-cell imaging was recorded for each of the three periods. **A** Endomitosis was the most prominent in Period 2 (2.5%) compared to Period 1 (0.4%) and Period 3 (2%). **B** Attempts to perform mitotic activity gradually recovered over time, from 4% at Period 1 to 11% at Period 2 then 20% at Period 3. **C** Mitotic level remained < 1% at Period 1 and 2, and significantly recovered to 8% at Period 3. **D** Frequency of DNV jumped from 0.5% at Period 1 and 2–5% at Period 3. **E** Cannibalism was observed at Period 1 (2%) and Period 2 (0.5%) but absent at Period 3. **F** Period 1 had 6% shedding activity that declined to 2% at Period 2 and 0.2% at Period 3. Mitosis are represented in blue; abnormal mitosis are in pink; failed mitosis are in hatched region and non-mitotic are in green. Statistical significance were estimate using GraphPad prism t-test to yield ***p < 0.0001; **p < 0.001; and *p < 0.01

When live-cell imaging of Case 1 cells was performed during their recovery from an anti-cancer drug administered as pre-operation adjuvant chemotherapy, endomitosis and attempts at mitosis began on days 7–9, and increased in frequency on days 10–13 (Fig. 4A, B). The rate of mitosis recovered extensively by day 18 accompanied by small decreases in endomitosis and larger decreases in failed mitosis (Fig. 4C). DNV formation and fusion of daughter cells occurred in during all three Periods: DNV frequency was similar to that of mitosis in Periods 1 and 2, but rose significantly in Period 3 (Fig. 4D, Additional file 1: Fig. S2). Even in Periods 1 and 2, some mother cells could produce six to eight daughter cells, some of which eventually entered into cell fusions (Additional file 1: Fig. S2). Amoeboid sequestration and shedding of cytoplasm were active in Period 1, less active in Period 2 and minimal in Period 3 (Fig. 4E, F), although most cells exhibited tailing and tunneling even in Period 3. Therefore, the diminution of mitosis, most severe at the onset of the primary culture gradually recovered through the three Periods. Each Period was dominated by one of the three different modes of proliferation, i.e., non-mitosis (green columns), abnormal/failed mitosis (pink columns), and mitosis (blue columns) (Fig. 4).

Non-mitotic proliferation was observed in the primary cultures of other cases including malignant meningioma, benign meningioma and solitary metastasis as well. Even though these cases were diagnosed as different types of tumors, their primary cultured cells all exhibited abnormal cellular behaviors to various extent. Case 4, 6 7, 8 and 70, displayed significant instances of tunneling (Additional file 4: Video S3B–F). Shedding was recorded in Case 3, 6, 7, 8, 9 and 70 (Additional file 3: Video S2C–H). Variation in number of daughter cells was a common occurrence in Case 3, 11 and 70 (Additional file 6: Video S5). Binary fission was noted in Case 4 and 70 (Additional file 5: Video S4B-C). Case 70 also exhibited cannibalism (Additional file 2: Video S1C). Regardless of whether the tumor was malignant, benign, or of a more aggressive type, all of these cases demonstrated aberrant cell behavior, mirroring the characteristics observed in Case 1 cells. Overall, aberrant cellular behaviors were much more prominent with tumors with extreme malignancy such as the two-times recurrent tumor of Case 1.

### Non-mitotic proliferation in cultures of HeLa and A549 cell lines

**Fig. 5:**
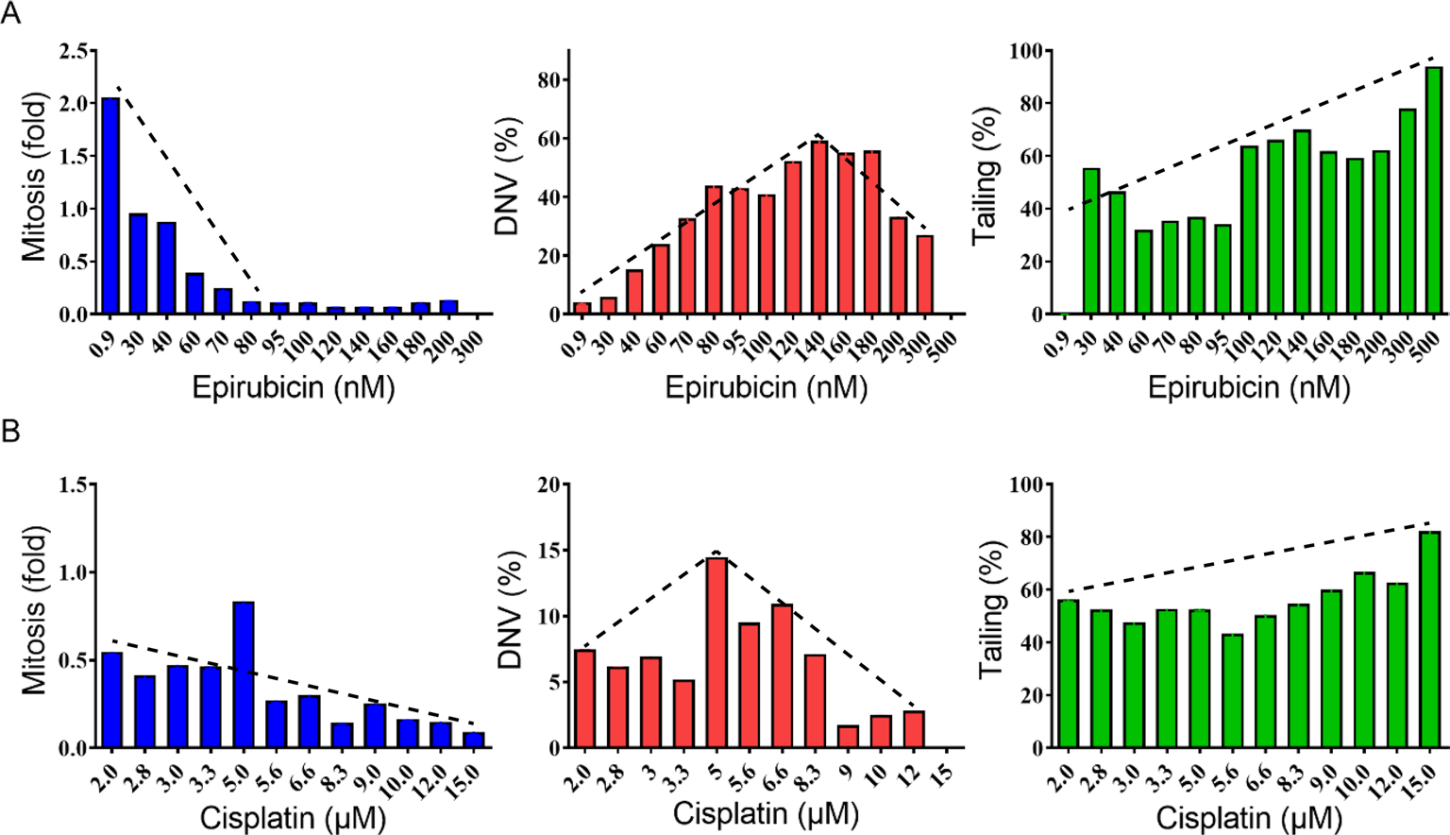
Abnormal mitotic and non-mitotic activities increased over increasing dosages of anti-mitotic drugs in cultured HeLa cells. HeLa cell cultures were treated with increasing concentrations of epirubicin (**A**) or cisplatin (**B**) as indicated on X-axis. **A** The mitosis level of HeLa fell in proportion to epirubicin dosage increase. The abundance of DNV in percentage under increasing dosages of epirubicin showed a reverse V-shape curve that reached peak level at 140 nM. Tailing increased along with doses and all cells were tailed at 500 nM. **B** Mitosis was most active at 5 μM of cisplatin then fell below 0.2-fold at higher doses compare to the 0 drug treatment. DNV showed a reversed V-shape curve that reached peak level at 5 μM. Tailing started appearing at 2 μM and gradually increased at higher dosage. Levels of mitosis, abnormal mitosis or non-mitosis in HeLa cell cultures were quantified by averaging at least two replicated experimental measurements. The dashed lines are trend-fittings to data on each panel illustrating the levels of mitosis (decreasing), abnormal mitosis (inversed V) or non-mitosis (increasing) along with increasing concentration of epirubicin (**A**) or cisplatin (**B**)

In order to determine how the aberrant cell-behavior manifest in the Case 1 primary-culture cells might differ from established human HeLa and A549 cancer cell lines, these two kinds of cells were treated with different dosages of cisplatin and epirubicin and monitored by live-cell imaging for 48 h. The IC_50_ dosages for HeLa and A549 were 4.3 µM and 12 µM respectively for cisplatin, and 190 nM and 160 nM respectively for epirubicin (Fig. 7A). For the HeLa cells, the rate of mitosis was elevated up to two-fold by 0.9 nM epirubicin, whereas the rate of cell tailing was increased substantially over a broad range of epirubicin. Treatment with low-moderate dosages of cisplatin enhanced both mitosis and DNA, while treatment with a broad range of cisplatin dosages caused a high rate of cell shedding (Fig. 5). On the other hand, for the A549 cells, both mitosis and DNV rates were sharply elevated at low dosages of epirubicin, while cell shedding was elevated by higher dosages of epirubicin. In a similar matter, cisplatin strongly induced mitosis at low dosages, DNV at medium dosages, and cell shedding at medium to high dosages. Moreover, the cells exhibited amoeboid sequestration at 8.3 µM (Additional file 7: Video S6A), amitotic cell division at 12 µM of cisplatin (Additional file 7: Video S6B), and double mitosis at 5 µM cisplatin or 20 nM epirubicin, where they underwent mitosis twice within 5 min (Additional file 7: Video S6C).

**Fig. 6:**
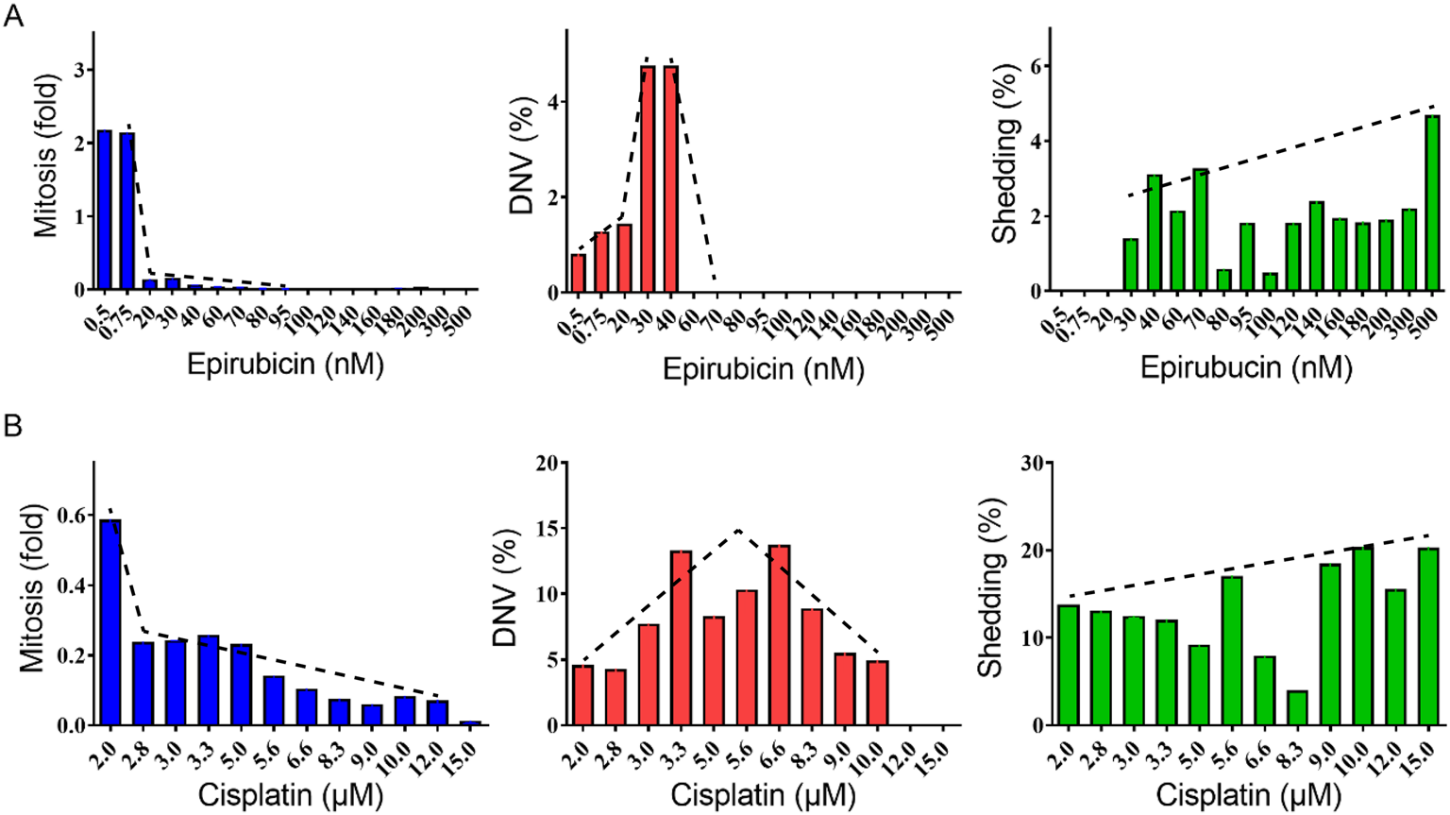
Abnormal mitotic and non-mitotic activities increased over increasing dosages of anti-mitotic drugs in cultured A549 cells. A549 cell cultures were treated with increasing concentrations of epirubicin (**A**) or cisplatin (**B**) as indicated on X-axis. **A** Mitosis was completely compromised at all doses except 0.5 nM and 0.75 nM. DNV only occurred at 40 nM or below with the highest percentage appeared at 30–40 nM of epirubicin. Shedding appeared at the 30 nM or above and remained at a relatively low level until epirubicin dosage reached 500 nM. **B** Mitosis gradually fell correlated with increased dosage of cisplatin. Relative abundance of DNV showed a reversed V-shape curve and reached the highest percentage at 3.3–6.6 μM. Levels of mitosis, abnormal mitosis or non-mitosis in HeLa cell cultures were quantified by averaging at least two replicated experimental measurements. The dashed lines are trend-fittings to data on each panel illustrating the levels of mitosis (decreasing), abnormal mitosis (inversed V) or non-mitosis (increasing) along with increasing concentration of epirubicin (A) or cisplatin (B)

Epirubicin exerted potent effects on the malignant A549 cells: mitotic rates were doubled by very low dosages of 0.5–0.75 nM (Period 1) and became fully suppressed at dosages above 20 nM (Fig. 6A). DNV increased with epirubicin dosage up to 30–40 nM (Period 2) but became suppressed at higher dosages. While tailing was not observed with the epirubicin-treated A549 cells, shedding of parts of the cell body occurred at 30–500 nM (Period 3). The epirubicin-treated A549 cells also showed amoeboid sequestration at 180–300 nM (Fig. 6A and Additional file 8: Video S7A).

In the cisplatin-treated A549 cells, mitotic rate declined to 60% of the untreated level at 2 µM, to 20% at 2.8–5.0 µM, and even more so at 5.6–15 µM (Fig. 6B). DNVs were readily observed at 2 µM cisplatin, and peaked at about 3.3 µM and 6.6 µM. Shedding was plentiful throughout the 9.0–15.0 µM range, and there was an example where the shed portion apparently recovered to form a new live cell (Additional file 8: Video S7B).

Relative to epirubicin-treated A549 cells, the cisplatin treated cells displayed more non-mitotic proliferations. For example, binary fission (Additional file 8: Video S7C) and amoeboid sequestration resulting in ‘cell-eating-cell’ was observed at 5.0–10.0 µM (Additional file 8: Video S7E). A special type of mitosis was also encountered in A549, where part of the cell did not participate in mitosis but joined one of the daughter cells afterwards (Additional file 8: Video S7D).

### Expressions of stem cell markers in cell lines upon recovery from drug treatments

**Fig. 7:**
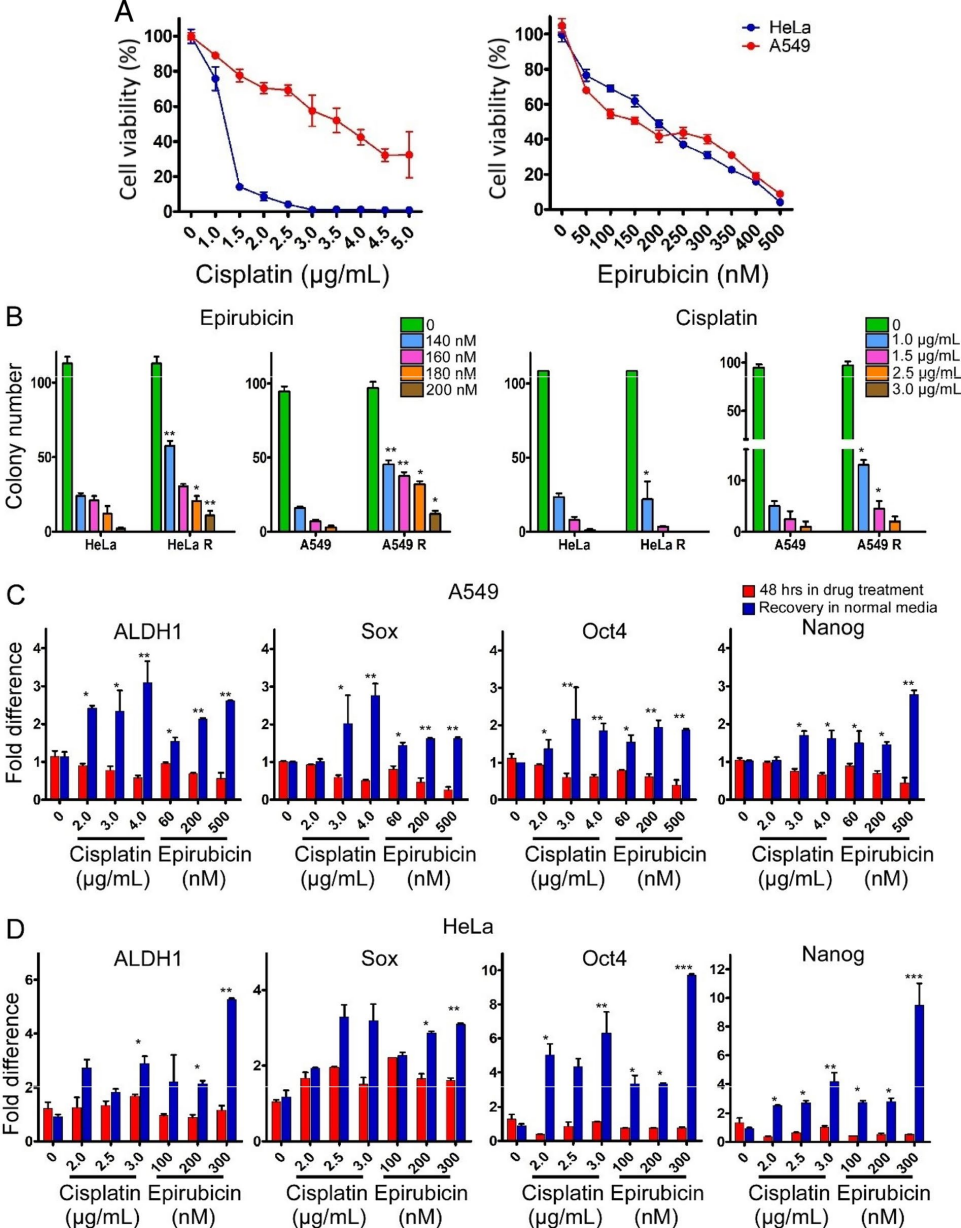
Elevated expression of stem-cell markers in cultured cell lines recovered from anti-mitotic drug treatments. **A** Viability of HeLa (blue circles) and A549 (red circles) cancer cells following treatment with cisplatin or epirubicin for 48 h. **B** Colony formation assay with HeLa and A549 cells. Cells were treated with various dosages of epirubicin or cisplatin for 48 h. Thereupon some cells remained as drug-treated cells in the drug-containing medium (HeLa and A549), while other cells were switched to drug-free medium (HeLa R and A549 R) and allowed to grow for 10 days before the colonies in each well were counted. Under epirubicin treatment both A549 and HeLa showed recovery in cell colonies. Under cisplatin treatment A549 retrieved cell colonies but HeLa failed to do so. **C**, **D** Expression of four different stem cell markers (ALDH1, Sox, Oct4, Nanog) in A549 (**C**) and HeLa (**D**). Red: 48 plus 48 h drug treatments; Blue: 48 h drug treatments and replaced with normal media for another 48 h to recover

Treatment of HeLa and A549 cells with epirubicin and cisplatin led to decreased cell proliferation measured by colony formation (Fig. 7B, red column). When these cells were incubated in fresh drug-free growth medium following 48 h of epirubicin or cisplatin treatment (Fig. 7B, blue column), they exhibited increases in colony formation. However, the cisplatin-treated cells recovered colony formation only when low dosages of cisplatin were employed for the treatment. These findings showed that the A549 cells could survive high dosages of the drug. Furthermore, expressions of the stem-cell markers Oct4, ALDH1, Sox2 and Nanog was studied on the drug treated cell (Fig. 7C, red column) and recovery cell (Fig. 7C, blue column). Lower dosage of cisplatin increases the expression of ALDH1 and Oct4 stem cell marker in A549 recovery cell. However, when exposed to higher dosages, all markers exhibited a significant elevation. Conversely, in the HeLa cell line, ALDH1, Oct4, and Nanog demonstrated heightened expression specifically under higher cisplatin dosages (Fig. 7D). ALDH1, Sox, Oct4 and Nanog were significantly increased in recovery cell at all the doses in both cell line (Fig. 7C, D).

## Discussion

**Fig. 8:**
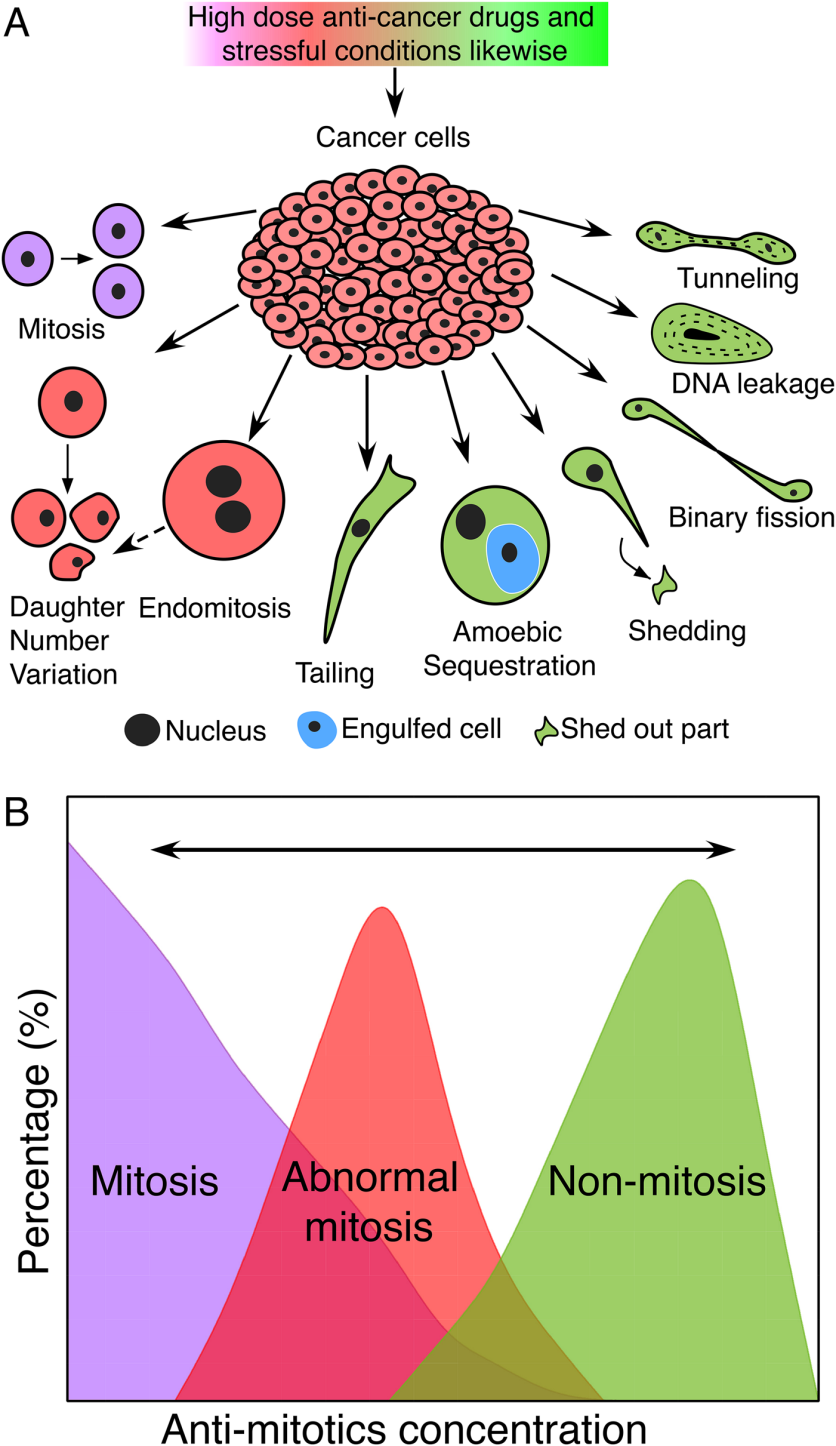
Graphical illustration of the three modes of cell proliferation, mitosis, abnormal mitosis, and non-mitosis. **A** Cellular behaviors under the three modes of proliferation. Purple: Mitosis; Pink: Abnormal mitosis; Green: Non-mitosis; **B** Reversable transitions, indicated by two-headed arrows, from mitosis, abnormal mitosis to non-mitosis or vice versa, under varying dosages of anti-cancer drugs. This hypothetical reversible transition may also apply to other stressful microenvironmental conditions, such as acidic environment

Based on observations reported herein, we hypothesize that cancer cells may adopt three alternative modes of proliferation. Besides regular mitosis and abnormal mitosis comprising mainly DNV and endomitosis, non-mitotic cellular changes such as tailing, shedding of cytoplasm, TNT, cannibalism and binary fission also became prominent in both primary cultures of malignant tumors such as HPC (Case 1), meningioma (Cases 3 and 6), metastatic brain cancer (Case 7), and the HeLa and A549 cell line cultures in the course of treatments by high-dosage cisplatin and epirubicin (Figs. 4, 5, 6, 8).

Live cell imaging revealed that Case 1 malignant tumor cell population contained numerous MNCs ranging from bi-nucleate to octa-nucleate cells, and MNC cells were reported to be largely chemo-resistant with the ability to generate clonal orthotopic metastatic tumors [26]. Mitosis of Case 1 cells gave rise to DNVs as recently described by us for the HeLa and HepG2 cell lines, with a mother cell generating multiple daughter cells, some of which fused together subsequently to form MNCs [20, 21]. The frequencies of DNVs and endomitosis as abnormal forms of mitotic proliferation increased over time during culture of the cells (Fig. 4A, D). In contrast to these aberrations of the malignant Case 1 tumor cells, the benign Case 2 tumor cells were devoid of MNCs. These findings confirmed the suggestion that DNV represented an activated SRC gene-dependent device where the malignant cancers can generate a regular supply of extra-malignant MNCs [22]. Since the daughter-cell fusions in DNV lead to the production of extra-malignant cells, inhibition of DNV-dependent cell fusions could represent a useful therapeutic approach. For this purpose, the yield of MNCs can be assayed based on the count of unfused daughter cells, and compounds such as 5-fluorouracil and wogonin were found to inhibit cell fusions [20, 21].

Non-mitotic behaviors were encountered in cell populations that displayed an array of striking morphological and behavioral changes seldom encountered in eukaryotic cells. They contained a large number of willow-shaped cells with tailing, tunneling nanotubes, amoeboid sequestration and shedding of cytoplasm. They also engaged in binary fission-like behavior with amitotic cell divisions that did not go through observable mitosis [12]; or cannibalism swallowing of nearby cells and debris [27]. Non-mitotic proliferation in the form of binary fission, along with amoeboid sequestration, shedding, tunneling and nucleic DNA leakage, was evident in the Case 1 cells when mitosis was inhibited by pre-operation and hence pre-culture chemotherapy (Fig. 3). Nucleic DNA leakage was more frequent in the malignant Case 1, Case 3 and Case 5 cells compared to the relatively benign Case 2, Case 4 and Case 6 cells, clearly indicating that occurrence of nucleic DNA leakage was related to a chromosomal instability which enhanced cell malignancy and triggered the metastasis of cells from a primary tumor to form distant tumors [28]. However, non-mitotic proliferation (green columns in Fig. 4) subsided when mitosis (blue columns in Fig. 4) was restored upon culturing the cells in an anti-cancer drug-free medium. The malignant Case 1, but not the benign Case 2, showed a high level of multi-nucleation (Fig. 2A, B) along with the presence of non-mitotic proliferation (Figs. 3, 4).

The morphological and cell behavioral changes were not limited to HPC; abnormal and non-mitotic proliferations were also observed with other types of malignant tumors in primary cell cultures. This was recorded by the video of live cell images of Case 70, a malignant case of brain metastatic tumor that differed from HPC Case 1 but demonstrated similar abnormality to the Case 1 cells including amoeboid sequestration, shedding, DNV and binary-fission like division.

Notably, the familiar human cancer cell lines HeLa and A549 were likewise susceptible to various types of abnormal cell behavior, collectively referred to as non-mitotic proliferative behavior in the present study, under chemotherapeutic treatment of either HeLa or A549 cells with high dosages of cisplatin or epirubicin (Figs. 5, 6). Therefore, both malignant tumors and cancer cell lines exhibited non-mitotic proliferation under stressful culture conditions. Previously, cannibalism was shown to be upregulated in chemotherapy-induced senescent cancer cells [11], and oral squamous carcinoma cells exhibited cannibalism under stressful conditions of low nutrient supply and acidic microenvironment [27]. When the HeLa and A549 cell were treated with cisplatin or epirubicin, mitotic events remained prominent at low doses or neutral pH, but the cells exhibited non-mitotic activities of cannibalism, shedding, cell tailing, debris eating and division without mitosis at high drug doses. (green columns in Figs. 5 and 6).

These findings validated our hypothesis that, under conditions of low drug dosages, the cells could carry out normal mitosis (Period 1). However, as the dosages were raised, mitotic activity diminished giving way to abnormal mitosis (Period 2). With further elevation of dosages, all forms of mitosis ceased entirely, and non-mitotic behaviors became dominant (Period 3). As indicated by earlier research, chemotherapy-induced senescent cancer cells were found to engulf other cells as a survival strategy [29, 30], and it was noted that the formation of tunneling nanotubes in MCF-7 breast cancer cells could promoted cancer cell survival against 5-fluorouracil [31].

Interestingly, the recoveries of HeLa and A549 cells from anti-cancer drug treatments (Fig. 7A, B), were accompanied by enhanced expression of the stem cell markers ALDH1, Sox2 and Nanog (Fig. 7C, D), which suggests a possible relationship between non-mitotic division and cancer stem cells in tumor recurrence [32–34].

Mechanistically, the occurrence of DNV was traceable to the activated SRC gene [20, 21], and it was suggested that homotypic cell cannibalism, regulated by the nuclear protein 1, opposed metastasis in pancreatic cancer [35]. Moreover, alterations in cell shape through formation of tunneling and tailing, guided by oncogene activity, were associated with the development of malignant tumors [36–40]. Such dependence of non-mitotic behavior on the malignancy of the cells was consistent with the likely involvement of oncogene. In the case of the HeLa and A549 cells, the expressions of the stem cell markers ALDH1, Sox, Oct4 and Nanog were enhanced following drug treatment, as indicated by the increased fraction of recovering cells (blue columns) compared to the cells under treatment (red columns) in Fig. 7. This difference between the red and blue columns was particularly pronounced in the case of Oct4 and Nanog in the HeLa cells, in agreement with the enrichment of Oct-positive and Nanog-positive stem cells in chemoresistance in cancer cells [41, 42].

## Conclusion

In conclusion, the present study found that increases in anti-cancer drug dosages led to a reduction in regular mitosis and enhancement of abnormal mitosis in malignant tumor cells, and further increases in dosages resulted in the display of non-mitotic proliferation such as binary fission, which was typical of prokaryotic cell division, suggestive of cell level atavism. It is noteworthy that malignant tumor cells generally exhibited non-mitotic behaviors, especially when under high anti-cancer drug dosages, which were rarely seen with benign tumor cells as far as in this study. The present findings therefore suggest that the malignant cells could resist total killing by high drug dosages through a reversable transition from normal mitotic to abnormal mitotic and non-mitotic proliferations (Fig. 8). Insofar that such transitions might reinforce the drug resistance of malignant tumors, in-depth investigation of the causes and therapeutic implications of the abnormal mitosis and non-mitosis modes of cell proliferation in various types of malignant cancers, as well as their relationships with the cancer stem cells over expressing markers such as Oct14 and Nanog, could be important to the development of more effective anti-cancer therapies. In addition, abnormal mitotic and non-mitotic cellular behaviors as indicators of tumor malignancy should be further explored to complement current pathology diagnosis criteria.

## Materials and methods

### Case description

HPC Case 1 was a 46-year-old male patient who was diagnosed with a right frontal HPC in October 2016 and treated with chemotherapy. Subsequent by first recurrence occurred on 4 June 2017, and second recurrence on 18 May 2020. The Case 1 tissue specimen was cultured during the second recurrence. HPC Case 2 was a 32-year-old female who recovered after chemotherapy. Case 3 was an 84-year-old male diagnosed with parietal lobe atypical meningioma. Case 4 was a 36-year-old female diagnosed with convexity typical meningioma. Case 5 was a 48-year-old female diagnosed with frontal lobe atypical meningioma. Case 6 was a 71-year-old male diagnosed with frontal lobe meningioma and borderline atypia. Case 7 was a 59-year-old female diagnosed with solitary metastasis of breast cancer (For summary see Additional file 1: Table S1). All the patients gave informed consent for the examination of their tissues, and the study protocol was approved by the ethics committee of the Prince of Wales Hospital of Hong Kong.

### Immuno-staining of tumor samples

Tumor samples retrieved from the patients were stained with H&E staining. Antibodies for STAT6, CD34, EMA and Ki67 were used on formalin fixed, paraffin embedded whole sections and tissue microarray slides. Distribution of staining was scored as: 0 (no staining), 1 + (1–25%), 2 + (26–50%), 3 + (> 50%). Intensity was scored as weak, moderate or very high. The Ki67 proliferation index is assessed by examining the hotspot areas (regions with highest labelling) in a tumor section and counting the number of tumor cells with positive nuclear stain out of all tumor cells in the hotspot histological fields.

### Primary cell cultures

Establishment of primary tumor cell culture from surgical specimen was carried out as described [28]. Tumor tissue was cut into small pieces and minced in Dulbecco’s modified Eagle medium (DMEM) containing fetal bovine serum (FBS), 100 IU/mL penicillin, 100 mg/mL streptomycin, and 2 mM L-glutamine. After digestion with collagenase and lipase for an hour at 37 °C, the cells were cultivated in DMEM growth medium supplemented with 10% FBS (Gemini Bio-Products, Sacramento, CA) under a humidified atmosphere of 5% CO_2_ at 37 °C, and growth medium was changed twice a week.

### Microscopic imaging analysis of primary and cell line cultures

Cells were seeded on 12-well plates at a density of 1 × 10^4^ cells per well. Their morphology, ploidy, mitotic, non-mitotic events consisting of shedding, cannibalism, tunnelling and binary fission, and abnormal mitosis consisting of DNV and endo-mitosis were monitored by time-lapse phase contrast photographs from twenty different positions of the well for 5-min increments for 5 days at 10X magnification using the CD7 cell discoverer. To study the leakage of nucleic acid materials into the cytoplasm and relevant cellular morphological changes such as nano-tubing formation between cells, the cells were seeded in DMEM growth medium supplemented with 10% FBS and stained with Hoechst 33342 (20 mM, Thermofisher) for 10 min at 37 °C. Afterwards, the staining medium was replaced by fresh medium, and cells were monitored using the CD7 cell discoverer for 24 h. Images were recorded from twenty different position at intervals of 8 min over the course of 24 h.

For multinucleation study, cells were seeded into 6-well plates at a density of 1 × 10^5^ per well in DMEM growth medium supplemented with 10% FBS and stained with Hoechst 33342 (20 mM, Thermofisher) for 10 min in an incubator at 37 °C. After 10 min, staining medium was replaced by DMEM growth media supplemented by 10% FBS and placed under the Nikon ECLIPSE T*i* microscope. For study, twenty positions were photographed for each case and all the cells under each view were counted manually using Image J software.

### Quantification of non-mitotic, abnormal mitotic and mitotic events

All cells were manually traced and quantified using the ImageJ software. Production of non-mitotic events (cannibalism, shedding, tailing, nucleic DNA leakage, tunnelling, binary fission, cell bursting), and mitosis was quantified by dividing the total number of events observed in the twenty positions by the overall number of cells present across all twenty positions. DNV percentage was estimated by dividing the total count of DNVs across the twenty positions by the total number of mitotic events observed across all twenty positions.

### Cell line cultures

The human HeLa cervical adenocarcinoma and A549 lung adenocarcinoma cell lines were obtained from American Type Culture Collection (ATCC, Manassas, VA, USA), and cultivated in DMEM growth medium supplemented with 10% FBS (Gemini Bio-Products, Sacramento, CA) under a humidified atmosphere of 5% CO_2_ at 37 °C. The drug treatment medium consisted of DMEM growth medium with varied dosages of cisplatin or epirubicin from Sigma‐Aldrich (St Louis, USA). Stock solutions of cisplatin and epirubicin were made up in water at 5 µM and dimethyl sulfoxide (DMSO) at 20 µM respectively.

### Drug treatment viability assay

Cell cytotoxicity by cisplatin and epirubicin was measured in vitro using the MTT ([3-(4,5-dimethylthia-zol-2-yl)-2,5-diphenyltetrazolium bromide]) assay. The HeLa and A549 cells were cultivated in DMEM growth medium overnight to a monolayer on 96 well plates at a density of 1 × 10^3^ cells per well. Thereupon the DMEM growth medium was changed to medium containing varied doses of drug for 48 h under a humidified atmosphere of 5% CO_2_ at 37 °C. For each treatment five replicates were used. After 48 h, cells were treated with 20 µl of 5 mg/ml MTT solution for three hours. Dimethyl sulfoxide was added to each well to solubilize the formazan, which was measured at 570 nm and compared with vehicle (i.e. DMSO) containing control cells. All experiments were repeated three times with a time gap of three days.

### Colony formation assay

Cells were seeded on 6-well plates for overnight at a density of 3 × 10^2^ cells per well. Thereupon they were treated with different concentration of cisplatin (3.3, 5.0, 8.3 or 10.0 µM) or epirubicin (140, 160, 180 or 200 nM). After 48 h, some of the wells were replaced by drug-free medium, and designated as ‘recovery’ wells (R), while the remaining wells were left in drug-containing medium. After 10 days, colonies were counted in all the recovery wells (R) and drug treated cells.

### Real time PCR for stem cell markers

HeLa and A549 cells were seeded on 6-well plates at a density of 1 × 10^5^ cells per well. The wells were treated with varied doses of cisplatin or epirubicin. After 48 h, the drug-containing medium in some wells was replaced by drug-free medium to form the recovery samples, while the drug-containing medium in the remaining wells remained unreplaced to form the drug-treated samples. Both types of cells were incubated for another 48 h. Thereupon, TRIzolTM (Invitrogen Corp., CA) was added to each well to prepare the total RNA extract. The mRNA contents of different stem-cell markers in the total RNA extract were estimated by means of real-time PCR (RT-PCR) using the QuantiTect® reverse transcription kit (Qiagen), followed by determination of the cDNA content with the FastStart Universal SYBR Green Master (Roche) on the LightCycler PCR System (Thermo Fisher Scientific). RNAase P and albumin mRNAs were employed as references. The stem cells markers employed included: ALDH1 (forward: 5′-TTCTGACTGTCACCTGGAGCCT-3′, reverse: 5′-GGCTGGGCTTGTAAGATGCCT-3′); NANOG (forward: 5′-TACCTCAGCCTCCAGCAGAT-3′, reverse: 5′-CATCCCTGGTGGTAGGAAGAGT-3′); OCT4 (forward: 5′-CCTCCAGCAGATGCAAGAACT-3′, reverse: 5′-TCCCTGGTGGTAGGAAGAGT-3′); SOX 2 (forward: 5′-CAACGGCAGCTACAGCATGAT-3′, reverse: 5′-GTTCATGTAGGTCTGCGAGCT-3′); RNAse P (forward: 5′-CTAACAGGGCTCTCCCTGAG-3′, reverse 5′-CAGCCATTGAACTCACTTCG-3′); and Albumin (forward: 5′-AATGCTGCACAGAATCCTTGGT-3′, reverse 5′-TCATCGACTTCCAGAGCTGAAA-3′).

### Statistical analysis

The GraphPad Prism 5.0 software was used for all statistical analysis, and the results were presented as the mean value. Significance was determined using the Student t-test (two-tailed, unpaired/unequal variances), p < 0.05 was regarded as statistically significant.

### Supplementary Information

**Additional file 1: **Supplementary figures, tables and a list of videos.

**Additional file 2: Video S1**. Cannibalism in primary cultures: A. Cannibalism in Case 1 (0–24 s, scale bars = 20 µm); B. Cannibalism in Case 1 (24–33 s, scale bars = 20 µm); C. Cannibalism in Case 70 (33–39 s, scale bars = 20 µm); D. Two close-up cells are eating parts of each other in Case 1. The red, yellow, and blue arrows represent the three nuclei respectively, and the green and purple arrows indicate that the cells are eating parts of each other (39–51 s, scale bars = 20 µm).

**Additional file 3: Video S2**. Shedding in primary cultures: A. Shedding in Case 1 (0–52 s, scale bars = 50 µm); B. Shedding in Case 1 (52 s–1 min 17 s, scale bars = 20 µm); C. Shedding in Case 3 (1 min 17 s–1 min 26 s, scale bars = 20 µm); D. Shedding in Case 6 (1 min 26 s–1 min 37 s, scale bars = 20 µm); E. Shedding in Case 7 (1 min 37 s–1 min 47 s, scale bars = 20 µm); F. Shedding in Case 8 (1 min 47 s–1 min 59 s, scale bars = 20 µm); G. Shedding in Case 9 (1 min 59 s-2 min 8 s, scale bars = 20 µm); H. Shedding in Case 70 (2 min 8 s–2 min 28 s, scale bars = 20 µm); I. Shedding in Case 1 (2 min 28 s–2 min 37 s, scale bars = 20 µm).

**Additional file 4: Video S3**. Tunneling in primary cultures: A. Tunneling in Case 1 (0–14 s, scale bars = 20 µm); B. Tunneling in Case 4 (14–21 s, scale bars = 20 µm); C. Tunneling in Case 6 (21–41 s, scale bars = 20 µm); D. Tunneling in Case 7 (41–48 s, scale bars = 20 µm); E. Tunneling in Case 8 (48–55 s, scale bars = 20 µm); F. Tunneling in Case 70 (55 s-1 min 01 s, scale bars = 20 µm); G. Tunneling in Case 1 (1 min 01 s-1 min 11 s, scale bars = 20 µm); H. Tunneling in Case 1 (1 min 11 s-1 min 24 s, scale bars = 20 µm).

**Additional file 5: Video S4**. Binary fision in primary cultures: A. binary fision in Case 1 (0–12 s, scale bars = 20 µm); B. binary fision in Case 4 (12–31 s, scale bars = 50 µm); C. binary fision in Case 70 (31–42 s, scale bars = 20 µm).

**Additional file 6: Video S5**. Daughter Number Variation in primary cultures: A. Case 3 shows DNV formation (0–16 s, scale bars = 20 µm); B. Case 11 shows DNV formation and shedding (17–45 s, scale bars = 20 µm); C. Case 70 shows DNV formation (45–56 s, scale bars = 20 µm).

**Additional file 7: Video S6**. Non-mitotic behaviors in HeLa cell culture: A. Cannibalism in HeLa (0–20 s, scale bars = 20 µm); B. DNV formation in HeLa cell line (20 s-1 min 35 s, scale bars = 20 µm); C. DNV formation in HeLa (1 min 35 s-1 min 44 s, scale bars = 20 µm).

**Additional file 8: Video S7**. Non-mitotic behaviors in A549 cell culture: A. Cannibalism in A549 (0–1 min 14 s, scale bars = 20 µm); B. Shedding in A549 (1 min 14 s-1 min 23 s, scale bars = 20 µm); C. Binary fission in A549 (1 min 23 s-1 min 33 s, scale bars = 20 µm); D. DNV formation A549 (1 min 33 s-1 min 39 s, scale bars = 20 µm); E. Cannibalism in A549 cell line (1 min 39 s-2 min 28 s, scale bars = 20 µm).

## Data Availability

The datasets used or analyzed during the current study are available from the corresponding author upon reasonable request.
