# Supplementary material for: Non-mitotic proliferation of malignant cancer cells revealed through live-cell imaging of primary and cell-line cultures
Source: Cell Div. 2024 Feb 10;19:3. doi: 10.1186/s13008-024-00109-x (PMC10858565; doi:10.1186/s13008-024-00109-x)
Supplement: Supplementary file 1 — Additional file 1: Supplementary figures, tables and a list of videos. [file 13008_2024_109_MOESM1_ESM.docx]

**SUPPLEMENTARY FIGURES**


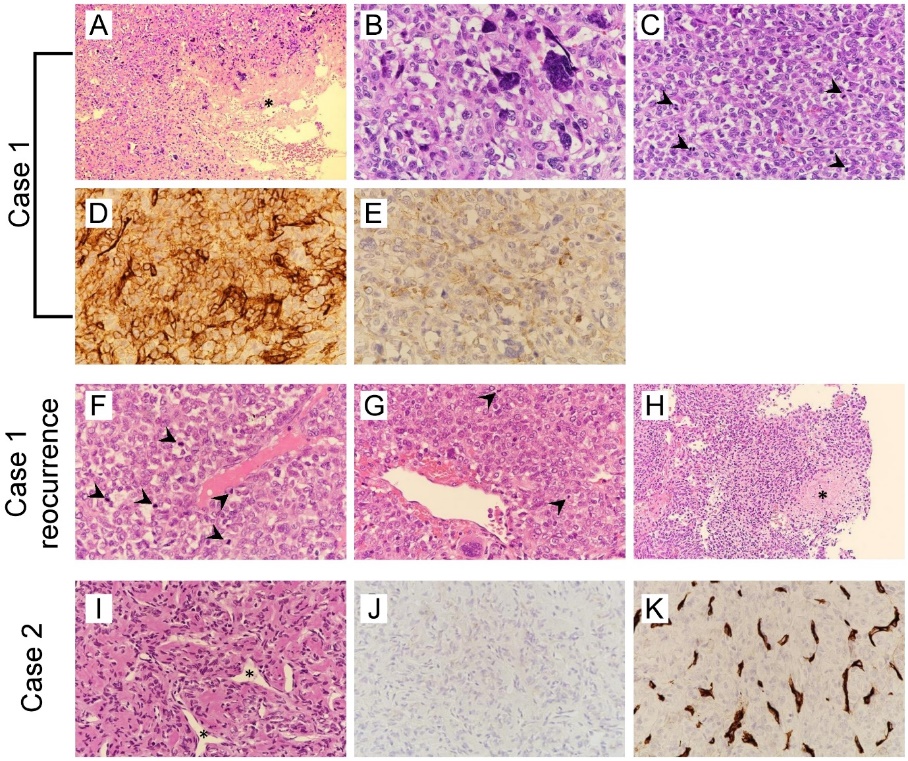


**Supplementary Figure S1. Distinction of Case 1 from Case 2 in histopathology.** Representative snapshots of H&E (A, B, C, F, G, H, I) and immuno-histochemical (D, E, J, K) staining of tumor tissue samples from Case 1 and Case 2, respectively. (A) Case 1 primary tumor showed foci of necrosis (asterisk) (200X). On high power examination, (B) the tumor cells demonstrated marked nuclear pleomorphism (400X), and (C) brisk mitotic figures (arrow heads) (400X). Immuno-histochemical staining showed (D) CD34 expression (400X), and (E) very weak expression of EMA (400X). Both the first recurrent (F) and second recurrent (G) tumors demonstrated diffuse hypercellularity, brisk mitosis (arrowhead) (400X); and (H) the second recurrent tumor showed necrotic foci (asterisk) (200X). Under high power field (400X), Case 2 showed (I) haphazardly arranged spindle cells with moderate cytological atypia, set in collagenous stroma intermixed with staghorn vessels (asterisk), lacking mitotic figures; and negative for (J) EMA and (K) CD34 staining.


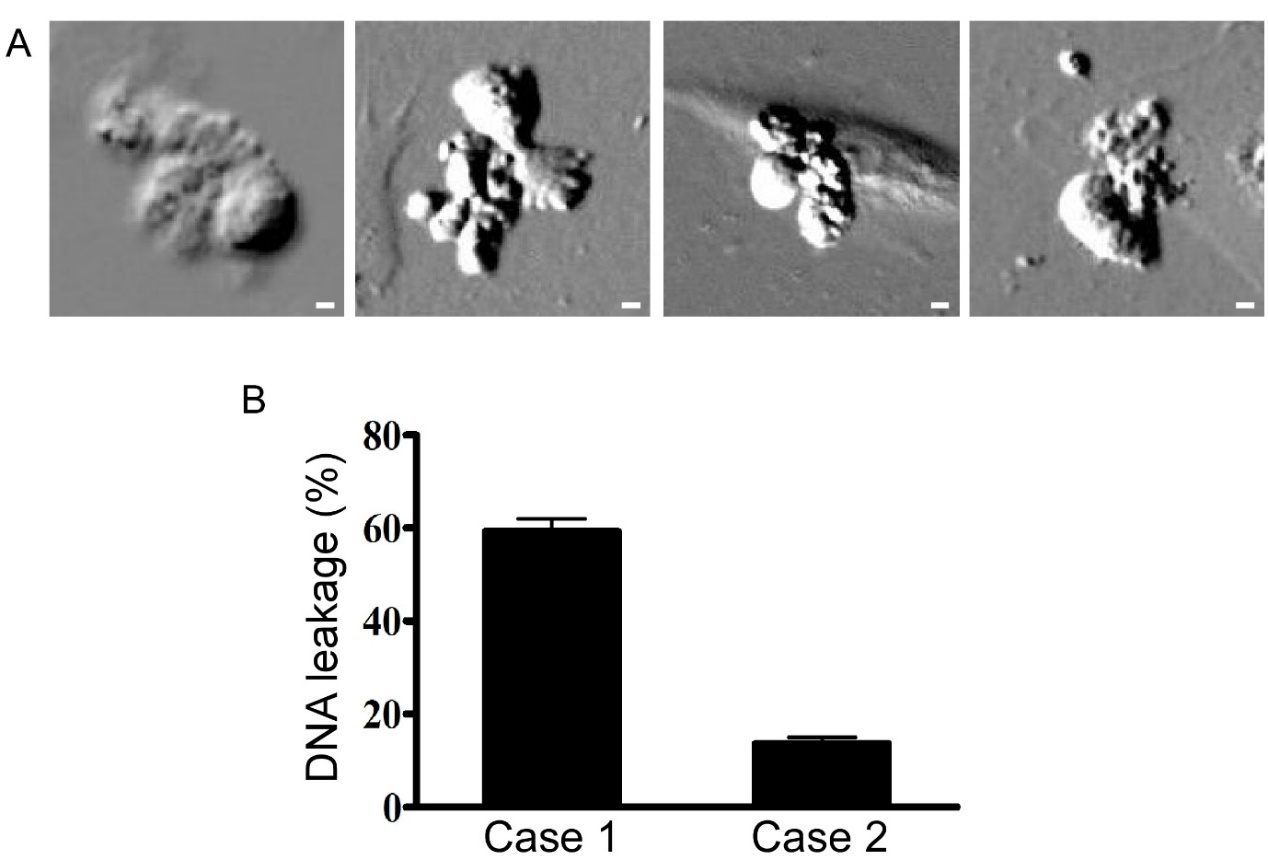


**Supplementary Figure S2**. **Daughter Number variation (DNV) and nucleic DNA leakage.** (A) Representative photo of DNV in Case 1 and (B) quantification of nucleic DNA leakage in Case 1 and Case 2, respectively. The scale bars in (A) are at 20 µm.

| **ID** | **Gender** | **Age** | **Diagnosis** |
| --- | --- | --- | --- |
| Case 1 | Male | 46 | Solitary fibrous tumor (hemangiopericytoma), 2^nd^ recurrence (exhibited non-mitotic behaviors in this study, referring to Videos 1, 2, 3 and 4) |
| Case 2 | Female | 32 | Tentorial solitary fibrous tumor (hemangiopericytoma) |
| Case 3 | Male | 84 | Parietal lobe atypical meningioma (exhibited non-mitotic behaviors in this study, referring to Videos 2 and 5) |
| Case 4 | Female | 36 | Convexity typical meningioma (exhibited non-mitotic behaviors in this study, referring to Videos 3 and 4) |
| Case 5 | Female | 48 | Frontal lobe atypical meningioma |
| Case 6 | Male | 71 | Frontal lobe meningioma, borderline atypia (exhibited non-mitotic behaviors in this study, referring to Videos 2 and 3) |
| Case 7 | Female | 49 | Parasagittal recurrent meningioma (exhibited non-mitotic behaviors in this study, referring to Videos 2 and 3) |
| Case 8 | Female | 73 | Meningioma (WHO Grade I) (exhibited non-mitotic behaviors in this study, referring to Videos 2 and 3) |
| Case 9 | Female | 53 | Malignant meningioma (Grade III) (exhibited non-mitotic behaviors in this study, referring to Video 2) |
| Case 11 | Female | 66 | Recurrent meningioma (exhibited non-mitotic behaviors in this study, referring to Video 5) |
| Case 70 | Female | 59 | Solitary metastasis (from breast) (exhibited non-mitotic behaviors in this study, referring to Videos 1, 2, 3, 4 and 5) |

**Table S1. Summary of patients with tissue specimen used in this study.**

**Table S2. Increase in cell length of hemangiopericytoma over extent time of primary culture.**

|  | **At the start of culture** | **After 30 days of culture** |
| --- | --- | --- |
| **Case 1** | **27 ± 4.56 µm** | **313 ± 180.08 µm** |
| **Case 2** | **300 ± 11.29 µm** | **399 ± 118.99 µm** |

**Videos**

**Video 1.** Cannibalism in primary cultures: A. Cannibalism in Case 1 (0-24s, scale bars = 20 µm); B. Cannibalism in Case 1 (24-33s, scale bars = 20 µm); C. Cannibalism in Case 70 (33-39s, scale bars = 20 µm); D. Two close-up cells are eating parts of each other in Case 1. The red, yellow, and blue arrows represent the three nuclei respectively, and the green and purple arrows indicate that the cells are eating parts of each other (39-51s, scale bars = 20 µm).

**Video 2.** Shedding in primary cultures: A. Shedding in Case 1 (0-52s, scale bars = 50 µm); B. Shedding in Case 1 (52s-1min 17s, scale bars = 20 µm); C. Shedding in Case 3 (1min 17s-1min 26s, scale bars = 20 µm); D. Shedding in Case 6 (1min 26s-1min 37s, scale bars = 20 µm); E. Shedding in Case 7 (1min 37s-1min 47s, scale bars = 20 µm); F. Shedding in Case 8 (1min 47s-1min 59s, scale bars = 20 µm); G. Shedding in Case 9 (1min 59s-2min 8s, scale bars = 20 µm); H. Shedding in Case 70 (2min 8s- 2min 28s, scale bars = 20 µm); I. Shedding in Case 1 (2min 28s-2min 37s, scale bars = 20 µm)

**Video 3.** Tunneling in primary cultures: A. Tunneling in Case 1 (0-14s, scale bars = 20 µm); B. Tunneling in Case 4 (14-21s, scale bars = 20 µm); C. Tunneling in Case 6 (21-41s, scale bars = 20 µm); D. Tunneling in Case 7 (41-48s, scale bars = 20 µm); E. Tunneling in Case 8 (48-55s, scale bars = 20 µm); F. Tunneling in Case 70 (55s-1min 01s, scale bars = 20 µm); G. Tunneling in Case 1 (1min 01s-1min 11s, scale bars = 20 µm); H. Tunneling in Case 1 (1min 11s-1min 24s, scale bars = 20 µm)

**Video 4.** Binary Fission in primary cultures: A. Binary fission in Case 1 (0-12s, scale bars = 20 µm); B. Binary fission in Case 4 (12-31s, scale bars = 50 µm); C. Binary fission in Case 70 (31-42s, scale bars = 20 µm)

**Video 5.** Daughter Number Variation in primary cultures: A. Case 3 shows DNV formation (0-16s, scale bars = 20 µm); B. Case 11 shows DNV formation and shedding (17-45s, scale bars = 20 µm); C. Case 70 shows DNV formation (45-56s, scale bars = 20 µm)

**Video 6.** Non-mitotic behaviors in HeLa cell culture: A. Cannibalism in HeLa (0-20s, scale bars = 20 µm); B. DNV formation in HeLa cell line (20s-1min 35s, scale bars = 20 µm); C. DNV formation in HeLa (1min 35s-1min 44s, scale bars = 20 µm)

**Video 7.** Non-mitotic behaviors in A549 cell culture: A. Cannibalism in A549 (0-1min 14s, scale bars = 20 µm); B. Shedding in A549 (1min 14s-1min 23s, scale bars = 20 µm); C. Binary fission in A549 (1min 23s-1min 33s, scale bars = 20 µm); D. DNV formation A549 (1min 33s-1min 39s, scale bars = 20 µm); E. Cannibalism in A549 cell line (1min 39s-2min 28s, scale bars = 20 µm)
